# Colorectal Cancer in Jordan: Survival Rate and Its Related Factors

**DOI:** 10.1155/2017/3180762

**Published:** 2017-03-28

**Authors:** Ghazi Faisal Sharkas, Kamal H. Arqoub, Yousef S. Khader, Mohammad R. Tarawneh, Omar F. Nimri, Marwan J. Al-zaghal, Hadil S. Subih

**Affiliations:** ^1^Field Epidemiology Training Program, Non-Communicable Diseases, Ministry of Health, Amman, Jordan; ^2^Jordan Cancer Registry, Ministry of Health, Amman, Jordan; ^3^Department of Public Health, Faculty of Medicine, Jordan University of Science & Technology, Irbid, Jordan; ^4^Primary Health Care Directorate, Ministry of Health, Amman, Jordan; ^5^Department of Nutrition and Food Technology, Jordan University of Science & Technology, Irbid, Jordan

## Abstract

*Objectives*. To estimate the survival rate of colorectal cancer (CRC) and determine its predictors among Jordanian patients who were diagnosed in the period of 2005–2010.* Methods*. This study was based on Jordan cancer registry. All CRC cases that were registered in cancer registry during 2005–2010 were analyzed using the survival analysis. The last date for follow-up was 1st Oct 2016.* Results*. A total of 3005 patients with CRC were registered during 2005–2010. The overall 5-year and 10-year survival rates for patients with CRC were 58.2% and 51.8%, respectively. The 5-year survival rate decreased significantly from 60.4% for the age <50 years to 49.3% for the age ≥70 years (*p* < 0.005). The 5-year survival rate was 72.1% for the localized stage, 53.8% for the regional stage, and 22.6% for the distant metastasis. In the multivariate analysis, the only factors that were significantly associated with survival were age, grade, stage, and location of tumor.* Conclusions*. The overall 5-year and ten-year survival rates for CRC were 58.2% and 51.8%, respectively. Increased age, poor differentiation, advanced cancer stage, and right-sided cancers were associated with lower survival rates. Screening strategies are needed for early detection of colon adenomas and colorectal cancer in Jordan.

## 1. Introduction

Colorectal cancer (CRC) is the third most common cancer in men and the second in women according to the latest GLOBOCAN worldwide estimation in 2012 [1]. About 55% of the cases are reported in the more developed countries. The highest rates were estimated to be in Australia/New Zealand (age standardized rate (ASR): 44.8 and 32.2 per 100,000 in men and women, resp.) and the lowest in Western Africa (ASR: 4.5 and 3.8 per 100,000) [1].

In 2012, CRC was estimated to cause 694,000 deaths (8.5% of total cancer deaths) with more deaths (52%) occurring in the less developed countries [1]. In the United States, CRC is the third most common cancer and the third leading cause of death due to cancer in both genders [2].

In Jordan, the ASR has increased from 12.6 per 100,000 in 2005 to 17.2 per 100,000 in 2010 [3]. According to the latest comprehensive cancer incidence report in 2012, CRC accounted for 11.3% of all newly diagnosed cases among Jordanians and ranked the second among all cancers in both genders. The overall crude incidence rate was 8.9/100,000 population (8.6 and 9.2/100,000 males and females, resp.). The overall ASR was 16.3/100,000 (15.9 and 16.6/100,000 males and females, resp.) [4]. According to Jordan mortality registry in 2013, neoplasms were the second leading cause of death (16.4% of total deaths), and cancer of small intestine, colon, rectum, and anus accounted for 2% of total deaths [5].

Survival studies have yielded different findings about the survival rate and prognostic factors between countries [6–11]. Different clinical and pathological prognostic factors have been proposed for CRC in the literature, including location of tumor, depth of invasion, tumor size, differentiation of tumor, tumor site, lymph node metastasis, and distant metastasis [9–12]. This study was conducted to estimate the survival rate of CRC and determine its predictors among Jordanian patients who were diagnosed in the period between 2005 and 2010.

## 2. Methods

This study was based on Jordan cancer registry. All CRC cases among Jordanians who were registered in Jordan cancer registry during the period of 2005–2010, with or without histopathology report, were included in the study and analyzed using the survival analysis. All cases died at the time of diagnosis; non-Jordanian patients and patients with multiple cancers were not included in this study.

For each registered patient, demographic and clinical characteristics were obtained from the Jordan cancer registry files and hospital medical records. Data about the type and stage of cancer were obtained from histopathology reports from governmental and private laboratories in addition to the medical records of hospitals. The histopathology type was categorized according to cancer site. The cancer stage was classified according to Surveillance Epidemiology and End Results staging rules into localized, regional, and distant metastasis and unknown stage. To identify the vital status of these patients, date of the last visit was obtained from the medical records. Besides, the vital status was ascertained from the Civil Registration Department using a unique national identification number. Only cancer related deaths were recorded as “death” in the survival analysis. The few non-cancer-related deaths, as ascertained from the Civil Registration Department, were considered as censored cases.

A period of observation was set for the included patients from the date of diagnosis to the last date of observation if the patient was alive (1st Oct 2016) and to the date of death if the patient died during the observation period. The follow-up end point was death from cancer. Duplication of patients was excluded through verifying national identification number, the use of full four digits' names, and matching the names and the addresses.

The Jordan cancer registry uses forms for data collection to collect data about sociodemographic characteristics including national identification number, name, age, marital status, and address and information related to cancer including histopathology, morphology, stage of cancer, location of tumor, date of diagnosis, date of last visit, and outcome. According to the registry, right-sided colon cancer is defined as cancer of the cecum and the ascending colon up to the hepatic flexure. Left-sided colon cancer comprises cancer of the splenic flexure and cancer in regions distal to the splenic flexure, including the rectum.

Ethical approval was obtained from the Institutional Review Board in Ministry of Health. Data were obtained from Jordan cancer registry through the standard data request form.

Data were analyzed using Statistical Package for Social Sciences Software (SPSS) version (20 IBM). Data were described using means and percentages. The overall survival was estimated using Kaplan-Meier product limit technique. Log-rank test was used to compare survival rates between groups. Cox-regression analysis was used to determine factors associated with the time to death. A *p* value < 0.05 was considered statistically significant.

## 3. Results

The total number of patients who were diagnosed with CRC and registered in Jordan cancer registry in the period of 2005–2010 was 3005 patients. The number of patients has increased from 370 in 2005 to 570 in 2010 with an increase of 54% during the five years' period. The median age at diagnosis was 62 years for males and 58 years for females. Male to female ratio was 1.3 : 1. The most commonly affected age group was 60 years and above (52.2%). The demographic and clinical characteristics of patients are shown in Table 1. Of all cases, 26.5% were localized, 23.1% were regional, 17.2% were advanced, and 32.3% were of unknown stage.

Histopathology of CRC showed that adenocarcinoma was the commonest morphology (85%). The morphology for the rest of patients was mucinous (colloid) adenocarcinoma (8.4%), other carcinomas (4.6%), carcinoids (0.7%), signet ring adenocarcinoma (0.9%), adenocarcinoma in adenomatous polyps (0.2%), and adenocarcinoma in villous adenoma (0.2%).

**Figure 1:**
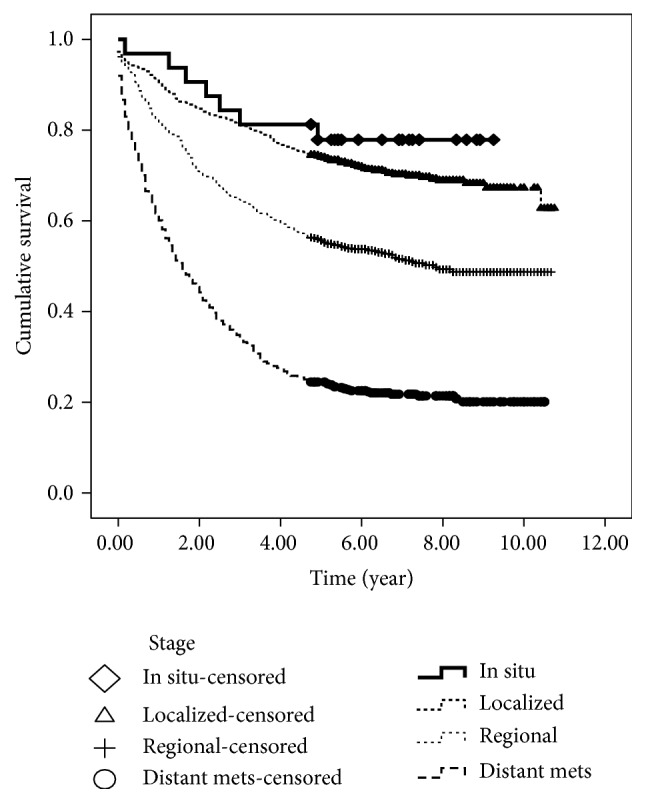
Survival rate for colorectal cancer according to stage.

The patients were followed up to the last date of observation if the patient was alive (1st Oct 2016) or to the date of death if the patient died of cancer. The median follow-up time was 5.2 years. The proportion of patients surviving each time interval and the cumulative survival are shown in Table 2. The overall 5-year and 10-year survival rates for CRC were 58.2% and 51.8%, respectively (Table 2). The 5-year survival rate decreased significantly from 60.4% for the age <50 years to 49.3% for the age ≥70 years (*p* < 0.005). The survival rate decreased significantly with the advanced stage of the disease (the 5-year survival rate was 72.1% for localized stage, 53.8% for regional stage, and 22.6% for distant metastasis) (Figure 1).  Table 3 shows the 5-year survival rate for CRC according to different prognostic variables including sex, age group, and site, morphology, grade, and stage of cancer.

Table 4 shows the multivariate analysis of factors associated with the hazard of death in Cox-regression analysis. The only factors that were significantly associated with death were age and grade, stage, and location of the tumor. The hazard of death increased significantly with increased age being the highest in age ≥70 years. The hazard of death was significantly higher for those with poorly differentiated cancer compared to those with well differentiated cancer (HR = 1.8). The hazard was much higher for patients whose cancer stage was regional (HR = 1.8) and those with distant metastasis (HR = 4.5) compared to those with localized cancer. The patients whose primary tumors originated on the right side of the colon had higher hazard of mortality compared to those whose tumors originated on the left side (HR = 1.3).

## 4. Discussion

Data on the survival analysis of CRC are scant in the Eastern Mediterranean countries including Jordan. Previous studies in other countries have reported variable CRC survival rates. In Asia, the highest survival rates were found in China and the lowest rate was reported in India [13–17]. The 5-year relative survival rate for patients who were diagnosed from 2003 to 2009 in United States was 64.9% [18]. This study showed that the overall 5-year survival rate for patients with CRC was 58.2%. This rate is higher than the reported rates from different countries in the Eastern Mediterranean region [8, 13, 16, 19, 20]. Various research studies from Iran have reported 5-year survival rates of CRC of 47% [16], 41% [8], and 61% [13], respectively. In another retrospective study in Iran, the 5-year survival rate was found to be 27.2% among 284 patients who were diagnosed with CRC between 2003 and 2008 [19]. In Saudi Arabia, the overall 5-year survival rate of CRC was 44.6% using the data from the cancer registry for the period of 1994–2004 [20]. The disparities in CRC survival between Eastern Mediterranean countries may be attributed to several factors including differences in socioeconomic status, stage at diagnosis, treatment, physician characteristics, and hospital factors. The better survival in Jordan compared with other countries in the region might be explained by the fact that cancer care in Jordan is more advanced in comparison to most neighboring countries, and the country hosts many local and western-trained physicians who can deliver various cancer treatment modalities [21]. Currently, the King Hussein Cancer Foundation and Center (KHCC) treats around 60% of cancer cases in Jordan. KHCC is a specialized tertiary hospital that provides all treatment modalities and services to Jordanian patients as well as other patients from neighboring countries. However, further studies are needed to examine the differences in CRC survival between these countries.

There is no significant difference in the survival between males and females in the univariate analysis and multivariate analysis. The lack of gender differences in survival rates was reported in some of the previous studies [13, 14, 16]. However, other studies had reported a lower 5-year survival rate in women [1, 22], that may be due to their increased incidence of right-sided cancer [22]. A systemic review reported that a higher proportion of women presents with right-sided colon cancer than men and the right-sided colon cancer is often at a more advanced stage at diagnosis [22]. In our study, there was no significant difference in the location of the tumor between men and women (*p* value = 0.098) and this might explain the lack of gender differences in survival rates.

The findings of previous studies concerning the effect of age were diverse. This study showed that the hazard of death increased significantly with increased age being the highest in age ≥70 years. This result was reported in other studies [8, 16] that showed that older patients had a poorer survival rate compared to younger patients. However, other studies [13, 23, 24] reported no difference in survival according to age. The contradictory results of previous studies on age may be due to inclusion of patients from single referral centers and poor adjustment for the effect of possible confounders.

Different clinical and pathological prognostic factors have been proposed for CRC in the literature, including location of the tumor [13, 16, 24, 25], tumor stage [26], differentiation of tumor [13], and surgical and distant metastasis [23]. The multivariate analysis using Cox-regression analysis showed that grade and stage were significant predictors of survival. The hazard of death was significantly higher for those with poorly differentiated cancer compared to those with well differentiated cancer. Moreover, it was much higher for patients whose cancers stage was regional and those with distant metastasis compared to those with localized cancer. A previous review study showed that CRC survival is highly dependent on stage at diagnosis, and the 5-year survival rate varied from 90% for localized stage cancers and 70% for regional cancer to 10% for distant metastatic cancer [25]. The study concluded that the earlier the stage at diagnosis, the higher the chance of survival.

This study showed a higher mortality hazard among patients whose primary tumors originated on the right side of the colon compared to those whose tumors originated on the left side. One study showed that tumors on the left side and right side have different underlying biological characteristic, different macroscopic properties, and different dominant pathways to relapse and hence may explain the differences in survival [27]. The higher hazard of mortality among patients whose primary tumors originated on the right side of the colon might have an implication on the diagnosis of CRC and on research to understand the differences in survival between right- and left-sided cancers.

Our study showed that the 5-year survival rate for CRC was 72.1%, 53.8%, and 22.6% for localized, regional, and distant stage, respectively. This finding is consistent with findings of many other studies [23, 26, 28]. The Thailand study showed that the 5-year stage specific survival rates for stages I, II, III, and IV CRC were 100%, 68%, 44%, and 2%, respectively [29]. In Saudi Arabia, the 5-year survival rate differed significantly according to the stage (63.3% for patients with localized disease, 50.2% for those with regional disease, and 14.7% for patients with metastases) [20]. The differences in the survival according to the stage are explained by the differences in the extent to which the cancer has spread and how many lymph nodes have been affected.

In a retrospective study in Iran on patients diagnosed with CRC from 2003 to 2008, multiple Cox-regression model revealed that survival rate has a significant relationship with other prognostic factors like the primary diagnosis method, income status, history of alcohol use, primary treatment method, and history of metastasis [19]. Data from Jordan cancer registry should be interpreted with caution. As many registries in the region, Jordan cancer registry does not collect information on other possible predictors of mortality such as occupation, level of education, economic status, and comorbidity. Therefore, our estimates of hazards ratio might be biased because of not adjusting for the effect of unmeasured variables.

In conclusion, the overall 5-year and ten-year survival rates for CRC were 58.2% and 51.8%, respectively. Increased age, poor differentiation, advanced cancer stage, and right- sided cancers were associated with lower survival rates. It is well established that CRC is one of those cancers that can largely be prevented by the early detection and removal of adenomatous polyps, and survival is therefore significantly better when colorectal cancer is diagnosed while being still localized. Screening strategies are needed for early detection of colon adenomas and colorectal cancer.

## Figures and Tables

**Table 1:** The demographic and clinical characteristics of 3005 patients diagnosed with colorectal cancer during the period of 2005–2010 in Jordan.

	*n*	%
Age (year)		
<50	748	24.9
50–59.9	691	23.0
60–69.9	891	29.7
70+	675	22.5
Sex		
Male	1,684	56.0
Female	1,321	44.0
Year at diagnosis		
2005	370	12.3
2006	439	14.6
2007	523	17.4
2008	549	18.3
2009	554	18.4
2010	570	19.0
Region		
North	557	18.7
Middle	2,319	77.7
South	109	3.7
Smoking		
Nonsmoker	2155	71.7
Smoker	850	28.3
Location		
Anus	48	1.6
Colon	1970	65.6
Rectum	987	32.8
Grade		
Well differentiated	164	5.5
Moderately differentiated	1,881	62.6
Poorly differentiated	277	9.2
Anaplastic	11	0.4
Unknown	672	22.4
Stage		
Localized	788	26.5
Regional	695	23.1
Distant metastasis	517	17.2
Unknown	972	32.3

**Table 2:** Life table of colorectal cancer cases diagnosed in the period of 2005–2010.

Interval start time (year)	Number at the beginning of the interval	Number of withdrawal cases	Number of patients exposed to risk	Number of deaths	Survival proportion	Cumulative survival proportion
1	2918	0	2918.0	519	82.2%	82.2%
2	2399	0	2399.0	298	87.6%	72.0%
3	2101	0	2101.0	174	91.7%	66.0%
4	1927	0	1927.0	140	92.7%	61.2%
5	1787	85	1744.5	86	95.1%	58.2%
6	1616	299	1466.5	53	96.4%	56.1%
7	1264	283	1122.5	29	97.4%	54.7%
8	952	261	821.5	19	97.7%	53.4%
9	672	277	533.5	7	98.7%	52.7%
10	388	214	281.0	5	98.2%	51.8%

**Table 3:** The 5-year survival rate according to different prognostic factors for 3005 patients diagnosed with colorectal cancer during the period of 2005–2010.

	*n*	The 5-year survival rate	*p* value
Age (year)			<0.005
<50	748	60.4	
50–59.9	691	58.2	
60–69.9	891	56.4	
70+	675	49.3	
Sex			0.141
Male	1,684	54.8	
Female	1,321	58.1	
Region			0.001
North	557	52.8	
Middle	2,319	57.5	
South	109	43.0	
Smoking			0.634
Nonsmoker	2155	56.9	
Smoker	850	55.1	
Location			0.800
Anus	48	53.4	
Colon	1970	57.0	
Rectum	987	54.7	
Grade			<0.005
Well differentiated	164	64.9	
Moderately differentiated	1,881	57.9	
Poorly differentiated	277	46.9	
Anaplastic	11	53.7	
Unknown	672	53.7	
Stage			<0.005
Localized	788	72.1	
Regional	695	53.8	
Distant metastasis	517	22.6	
Unknown	972	62.7	

**Table 4:** The multivariate analysis of factors associated with the hazard of death from colorectal cancer in Cox-regression analysis.

	HR	95.0% confidence interval for HR		*p* value
Sex (female versus male)	1.1	0.9	1.2	0.706
Age (year)				
<50	1.0			
50–59.9	1.1	1.0	1.3	0.143
60–69.9	1.4	1.1	1.7	0.001
70+	1.7	1.4	2.0	<0.001
Smoking (yes versus no)	1.1	0.9	1.2	0.253
Location				
Anus	1.0			
Colon	1.0	0.5	1.5	0.998
Rectum	1.2	0.7	1.7	0.711
Region				
North	1.0			
Middle	0.9	0.7	1.0	0.023
South	1.2	0.9	1.5	0.170
Grade				
Well differentiated	1.0			
Moderately differentiated	1.2	0.9	1.5	0.238
Poorly differentiated	1.8	1.4	2.3	0.001
Anaplastic	1.4	0.5	2.3	0.544
Unknown	1.3	0.9	1.8	0.051
Stage				
Localized	1.0			
Regional	1.8	1.6	3.7	<0.001
Distant metastasis	4.5	3.7	5.1	<0.001
Unknown	1.4	1.2	1.7	0.004
Location of tumor				
Left	1.0			
Right	1.3	1.1	1.6	0.013
